# Research on the Estimation Model of Electrical Parameters of Silver-Based Contacts Based on Surface Morphology

**DOI:** 10.3390/s25020312

**Published:** 2025-01-07

**Authors:** Chao Wang, Xiancheng Wang, Chengjun Guo

**Affiliations:** College of Science and Technology, Ningbo University, Ningbo 315000, China; 2211170018@nbu.edu.cn (C.W.); guochengjun@nbu.edu.cn (C.G.)

**Keywords:** surface morphology, surface texture characteristics, point cloud, parameter estimation model

## Abstract

The quality of surface morphology can reflect the electrical performance of silver-based contacts. Existing research on the correlation of morphological–electrical performance is based solely on empirical models from traditional visual inspections and only considers the impact of visually observable macro-textural features on electrical performance. However, the influence of micro-textural features on electrical performance should not be overlooked. This paper establishes a contactless surface morphology acquisition device based on a laser profilometer to address the assembly characteristics of contact components. Various original profile signals such as surface roughness, waviness, and surface form error are calculated using wavelet transformation, and a robust weight function is introduced to separate micro-textural features from macro-textural features. After the morphological parameters affecting electrical performance are quantified, the variation laws of single and composite morphological parameters on electrical performance are clarified. Parameter optimization iterations and parameter space distribution optimization are performed using a decision tree, and the optimized predictive model forecasts specific electrical parameter values. The predicted results are quantitatively evaluated, establishing evaluation metrics that reflect the errors and degree of fit between the model predictions and actual values from different perspectives. From the experimental results, the accuracy of the predictive model established in this study exceeds 97%.

## 1. Introduction

Silver-based contacts are important components for performing the disconnection function of low-voltage electrical connectors [1]. The electrical connection performance of the contacts can affect the reliability of the electrical connector and the entire electrical system, while the surface morphology quality of the contacts is one of the biggest influencing factors on electrical release performance [2]. Under working conditions, silver-based contacts are affected by mechanical factors [3] and electrical factors [4,5], resulting in irreversible material loss on the surface of the contact components. Changes in the surface morphology can affect the electrical contact performance of silver-based contacts after reaching a certain critical point.

Existing electrical degradation models for silver-based contacts are all predictive models based on empirical data. Different researchers have correlated structural parameters such as contact action frequency [6], relative slip amplitude [7], debris [8], and contact pressure [9] with the erosion patterns of surface quality and the stages of electrical parameter degradation through statistical and observational methods. Currently, some scholars have attempted to establish numerical models to characterize how changes in the morphology of the contact surface affect specific electrical parameters. Fouvry [10,11] connected the degree of surface wear and changes in contact resistance parameters using a transfer function, aiming to apply it to real electrical connector environments in the market. By combining coating structural micro-movement experiments, the stability of electrical connectors can be approximately calculated; however, this method only considers macroscopic defects in the surface coating and does not account for the impact of microscopic defects in the numerical model. Even introducing correction factors can lead to significant error accumulation. Keiji Mashimo [12] established a two-dimensional computational model of electrical connectors to study and analyze the mechanisms of debris and oxidation erosion on the surface of contact heads, using a finite element model to reveal the relationship between the migration loss of contact materials and changes in electrical performance. Some studies have also demonstrated that under different contact strokes, material elastoplasticity values, and contact pressures, certain electrical performance parameters can exhibit linear increases or decreases. Furthermore, after reaching certain thresholds, the mode of material loss can change significantly, resulting in nonlinear patterns of electrical parameter changes during this process, thereby establishing a numerical conversion model between morphological changes and electrical parameter variations [13,14,15].

This study is based on multiple contact surface erosion models, collects real-time morphological parameters of the contact surface, establishes a comprehensive morphological evaluation method, numerically characterizes the surface micro-morphological parameters that impact electrical performance during the degradation cycle of silver-based contacts, and builds an electrical parameter prediction calculation model. Ultimately, a system is constructed that can collect and calculate the characterization of electrical parameter changes in the micro-morphology of silver-based contacts in real time. The electrical parameter acquisition system based on precision numerical models can greatly enhance people’s control over the lifespan of electrical connectors, reducing production costs and unplanned maintenance.

## 2. Experimental Platform and Technical Scheme

This study uses a Keyence laser profiler as the device for acquiring microscopic morphology information of silver-based contacts. It achieves non-contact point cloud data acquisition through monocular laser scanning technology. The dataset includes the surface roughness, waviness, and surface profile signals of the objects being sampled. The model of the laser profiler used in this study is LJ-G5000 (Keyence, Osaka, Japan), with a preset X/Y scanning interval of 5 µm, a shooting frequency of 120/s, and a stepper motor-driven conveyor belt with a preset speed of 600 µm/s. To streamline data storage, modules for light intensity, color, and reflectivity are disabled to enhance real-time acquisition efficiency.

**Figure 1:**
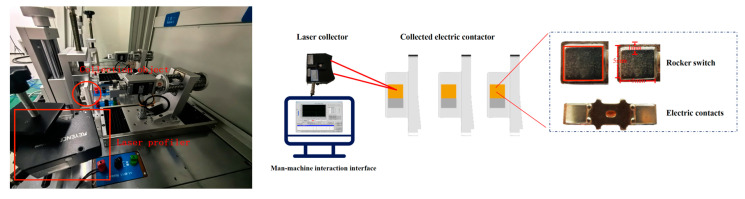
SystemPhysical devices and human–computer interaction interfaces.

**Figure 2:**
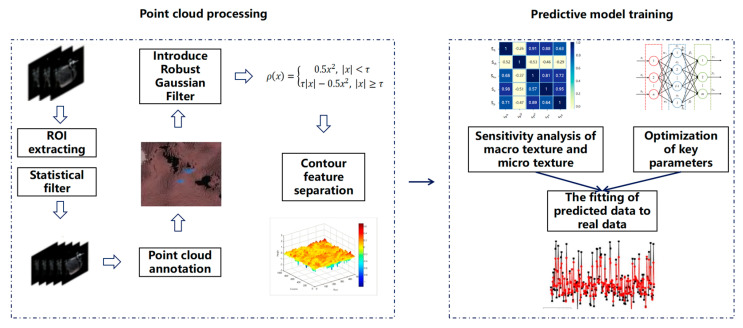
Technical solution display diagram of the electrical estimation.

After acquiring the point cloud dataset, it goes through three modules: point cloud filtering, texture signal extraction, and estimated model fitting to output simulated electrical values. Figure 1 is a technical illustration of this study, and Figure 2 is a schematic diagram of the experimental platform’s physical setup.

## 3. Point Cloud Preprocessing and Modeling

**Figure 3:**
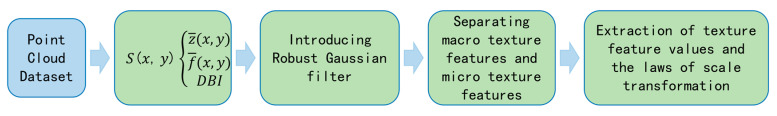
Point cloud dataset processing workflow.

The key to obtaining three-dimensional surface morphology parameters lies in correctly interpreting the point cloud dataset. This section analyzes and filters the collected point cloud data to restore the true surface morphology parameters as much as possible, minimizing the impact of measurement errors on subsequent electrical estimation models. The specific process is shown in Figure 3 for point cloud preprocessing.

First, geometric distribution indicators and texture features are extracted from the chaotic point cloud data, which entails deriving the correct geometric deviation solution S(x,y). The geometric deviation solution consists of three parts: height deviation value $\bar{z}(x_{i},y_{j})$, reference plane parameter value $\bar{f}\left(x,y\right),$ and error index DBI, as shown in Equation (1).

(1) $$S\left(x,y\right)=\bar{z}\left(x,y\right)-\bar{f}\left(x,y\right)-DBI$$

The Height deviation dataset $\bar{z}(x_{i},y_{j})$ is first calculated. The true value and the collected value of a certain point cloud coordinate are represented as $Z_{ij}$ and $M_{ij}$, as shown in Equation (2):

(2) z¯(xi,yj)=∑i=1m∑j=1nMij(x,y)−zij(x,y)212

The error evaluation index DBI [16] is introduced to determine further suppression of analytical errors, where $D_{j}$ represents the stability of the analytical calculations, as seen in Equation (3). $S_{i}$ is used to calculate the dispersion degree and can measure the dispersion of specific point clouds within a single dimension. $X_{ij}$ represents the *i*-th point cloud of the X-th data category, $S_{i}$ is the error, and $N_{i}$ is the number of data points in that dimension.

(3) Dj=1Ni∑i=1NiXij−Si212DBI=1Ni∑i=1Nimaxj≠1⁡z¯ij(x,y)+zij(x,y)αi−αj2

In the fitting of the reference plane and the actual plane, non-convergent analytical solutions inevitably occur. To eliminate the interference of this error on the amplitude parameters, the least squares method is used to obtain $\bar{f}(x,y)$ after removing the error term. First, assuming that the fitting equation of the initial reference plane is expressed as Equation (4), then the sum of squared deviations ɛ can be represented by the true data within any collected plane, as seen in Equation (5).

(4) $$\bar{f}\left(x_{i},y_{j}\right)=a+bx+cy$$

(5) $$\left\{\begin{array}{c} \frac{\partial \varepsilon }{\partial a}=\sum_{i=1}^{m}\sum_{j=1}^{n}\bar{z}(x_{i},y_{j})-a-bx_{i}-cy_{j}=0\\ \frac{\partial \varepsilon }{\partial b}=\sum_{i=1}^{m}\sum_{j=1}^{n}\bar{z}(x_{i},y_{j})x_{i}-ax_{i}-bx_{i}^{2}-cx_{i}y_{j}=0\\ \frac{\partial \varepsilon }{\partial c}=\sum_{i=1}^{m}\sum_{j=1}^{n}\bar{z}(x_{i},y_{j})y_{j}-ay_{j}-bx_{i}y_{j}-cy_{j}^{2}=0 \end{array}\right.$$

**Figure 4:**
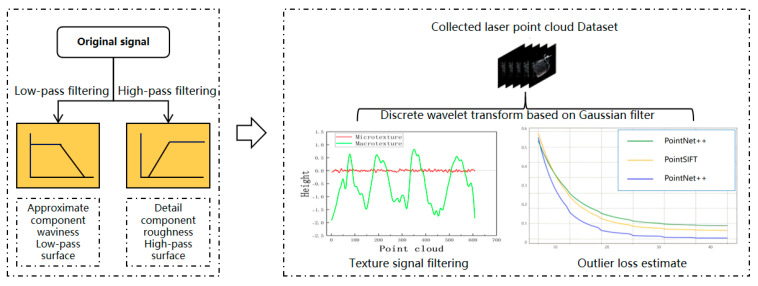
Introduction of a robust Gaussian filtering criterion for surface texture separation.

The geometric distribution values and texture feature values are calculated through the analytical model; however, during the parameter analysis of the point cloud, we can only ensure a reduction in analytical error and cannot guarantee that surface anomalies ($S_{p}$,$\;S_{v}$) generated during the collection process will not affect the results. In the boundary processing of the original scanning area, the quality of the collected point cloud dataset has a significant impact. When there are too many discrete points and edge anomalies interfering with the dataset, the separation estimation results will deviate accordingly as the distribution of interference points shifts. The defect distribution results estimated by the robust Gaussian filtering algorithm can ensure that the conditional domain of the computed results converges and maintains continuity, avoiding distortion of texture information caused by the influence of the dataset. The following introduces a robust weight function filter to enhance the accuracy and estimation efficiency of the separation algorithm in extracting macro- and micro-textures, as shown in Figure 4.

After the initial preprocessing of the three-dimensional point cloud dataset, the texture distribution characteristics and microscopic morphology parameters can be quickly and accurately fitted, requiring the extraction of various frequency signals such as surface roughness, waviness, and surface form error from the original point cloud dataset $z\left(x,y\right)$ [17]. The mixed macro- and micro-surface texture information is separated from the profile signal, and a mathematical expression for separating substrate defect features is established, along with a robust Gaussian filter that corresponds to the preset structural type. $r\left(x,y\right)$ represents the macro-surface roughness signal, while $w\left(x,y\right)$ is the low-frequency reference signal caused by surface roughness and shape errors.

(6) $$z\left(x,y\right)=r\left(x,y\right)+w\left(x,y\right)$$

The influence of the robust weighting function on the Gaussian filter is very high. In order to balance the final efficiency and anti-interference capability of the algorithm, this study uses the classic Huber function [18,19] as the filtering impact function for weighted estimation. The specific process is as follows:

(7) $$\rho \left(x\right)=\left\{\begin{array}{c} \;\;\;\;\;\;\;\;\;\;\;\;0.5x^{2},\;\;\;\left|x\right|<\tau \\ \tau \left|x\right|-0.5x^{2},\;\;\;\;\left|x\right|\geq \tau \end{array}\right.$$

In the above equation, τ is the noise sensitivity coefficient, and the background noise follows a Gaussian distribution, with τ = 1.345 chosen to ensure good estimation efficiency.

We construct a cost function for generalized maximum likelihood estimation to obtain the most suitable standardized residual $e_{i}$, where $\hat{\sigma }$ is the standardized deviation of the corrected residual, and 0.6745 is taken as the unbiased estimator correction for the normal distribution of residuals. We take the partial derivative of the cost function and set it to 0. Assuming $\varphi \left(\hat{e_{i}}\right)=\partial \left(\rho \left(\hat{e_{i}}\right)\right)/\partial \left(\hat{e_{i}}\right)$, $ᴪ\left(\hat{e_{i}}\right)=\varphi \left(\hat{e_{i}}\right)/\hat{e_{i}}$, the simplified calculation process is shown in Equation (8).

(8) $$\left\{\begin{array}{c} \frac{\partial J\left(h\right)}{\partial h}=\sum_{i=1}^{n}\frac{\partial \left(\rho \left(\hat{e_{i}}\right)\right)}{\partial \left(\hat{e_{i}}\right)}\cdot \frac{\partial \left(\hat{e_{i}}\right)}{\partial h}=0\\ \sum_{i=1}^{n}ᴪ\left(\hat{e_{i}}\right)\left(\frac{z_{i}-h}{b_{i}^{2}{\hat{\sigma }}^{2}}\right)=0\\ J\left(h\right)=\sum_{i=1}^{n}\rho \left(\frac{e_{i}}{b_{i}\hat{\sigma }}\right) \end{array}\right.$$

Power function is $w\left(\hat{e_{i}}\right)=ᴪ\left(\hat{e_{i}}\right)$, equivalence function is $W=diag\left(w\left(\hat{e_{i}}\right)\right)$, and coefficient matrix is $\hat{B}=diag\left(b_{i}^{2}{\hat{\sigma }}^{2}\right)$.

It is evident that the standard residual function W is influenced by the equivalent weight matrix *W*, meaning that the equivalent weight function is linked to the grid depth of the data to be estimated. To improve computational efficiency, re-weighting is performed before every node depth estimation, thereby optimizing the grid depth and simplifying the difficulty of estimating grid nodes.

(9) $$\left\{\begin{array}{c} A^{T}{\hat{B}}^{-1/2}W{\hat{B}}^{-1/2}\left(z-Ah\right)=0\\ {\hat{h}}^{\left(j+1\right)}={\left(A^{T}{\hat{B}}^{-1/2}W^{\left(j\right)}{\hat{B}}^{-1/2}A\right)}^{-1}A^{T}{\hat{B}}^{-1/2}W^{\left(j\right)}{\hat{B}}^{-1/2}ȥ \end{array}\right.$$

## 4. Morphological Parameter Optimization and Sensitivity Coefficient Extraction

By ranking the importance of sensitive parameters, the priority of individual morphological parameters is enhanced, completing the linear multi-feature correlation and sensitivity analysis of morphological parameters on electrical performance. An electrical characteristic self-calculation and estimation model is established using the existing decision tree model and custom optimized spatial parameters.

According to the geometric specification requirements in the relevant standard GB/T 43481-2023 [20], some morphology parameters that affect the electrical contacts are extracted [21]. From the contour signals collected in the previous section, specific amplitude parameter values are extracted as the benchmark morphological parameters for this study: $S_{q}$, $S_{sk}$, $S_{ku}$, $S_{a}$ and $S_{p}$. Table 1 shows the numerical characterization methods of different morphological parameters.

**Figure 5:**
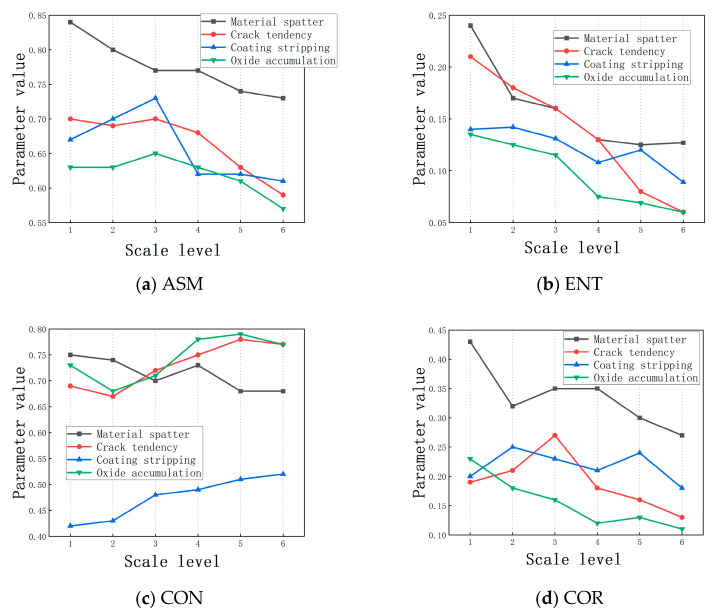
Analysis of the trend of scale transformation changes.

When processing multi-dimensional macro- and micro-texture image features, the results of different types of material loss methods based on Symlet discrete wavelet transform and gray level co-occurrence during texture image extraction are shown in Figure 5. This section separately presents the extraction efficiency of texture feature values and transformation scale levels in terms of ASM (angular second moment), ENT (entropy), CON (contrast), and COR (correlation). The x-axis represents the scale level, while the y-axis shows the final feature values under the corresponding scale transformation. As the scale level increases, ASM and ENT features as well as CON features exhibit local fluctuations but an overall downward trend, indicating that the texture distribution tends to become uniform and regularly computable. In contrast, contrast analysis shows an opposite trend, suggesting that the more drastic the scale transformation, the clearer the distribution of grooves in the textured surface image. This pattern aligns with the initial model design.

**Figure 6:**
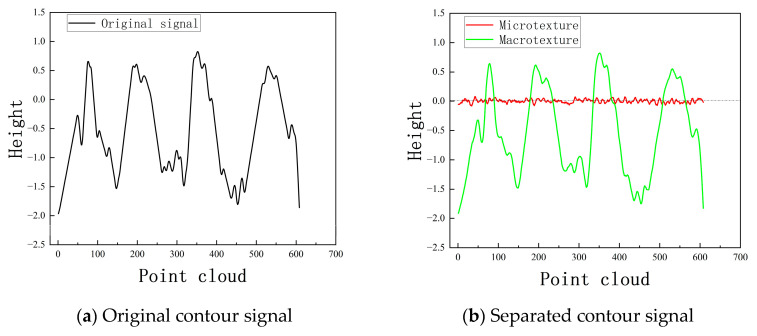
Contour signal separation results based on robust Gaussian filtering.

The collected original contour signal is displayed in Figure 6. From the figure, it can be seen that the macro-texture and micro-texture are mixed. If the contour signal is processed and analyzed directly, it is difficult to determine the impact of electrical performance in a certain area because it cannot be known whether that area is suppressed or enhanced, resulting in lower accuracy in the final electrical parameter estimation. Using a robust weight function to strip the original signal data Z from the scanned area, the midpoint of the two maximum value anomaly points S is selected as the center point for estimation, reducing the boundary effect on the filtering of the three-dimensional point cloud data. Figure 6a shows the selected original contour signal, and Figure 6b shows the stripped macro-morphology signal and micro-morphology signal.

**Figure 7:**
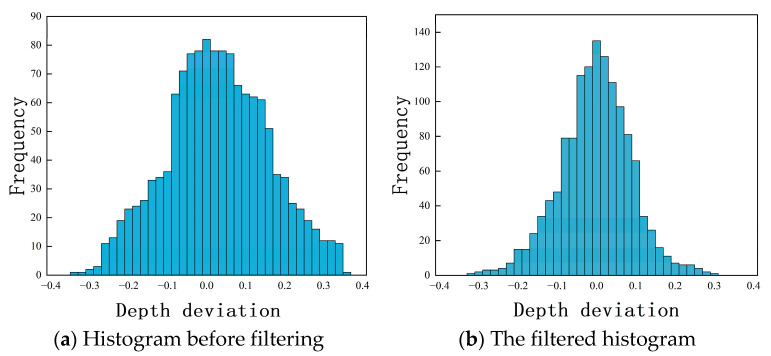
Verification of robust Gaussian filtering capability.

To verify the robust weight function filtering capability of the constructed model, the same segment of distorted dataset was used for outlier detection with a filtering model that did not incorporate the robust weight function. Figure 7 shows the structural separation errors with and without the integration of robust weight function filtering. It is evident that the error elimination capability significantly improved after introducing the weight function.

**Figure 8:**
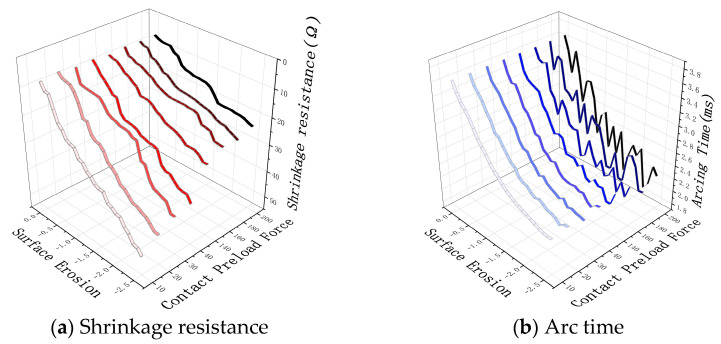
Degradation pattern of electrical performance under different pre-tightening forces. The darker the lines in the figure, the greater the pre-tightening force applied.

The installation structure of the electrical contacts will have a certain preload. If the preload is greater than or less than a certain threshold, it will accelerate the erosion behavior of the electrical contacts. As shown in Figure 8a, when the preload is below 140 mN, the shrinking resistance will accelerate degradation; in Figure 8b, when the preload is less than 150 mN, the fluctuation of the arcing time will increase. In order to prevent the preload from affecting the acquisition of the sensitivity coefficient in degradation experiments, it is necessary to choose an appropriate range of preload. After comprehensive consideration, this study sets the preload at 140 mN as the basic data for the degradation experiment.

**Figure 9:**
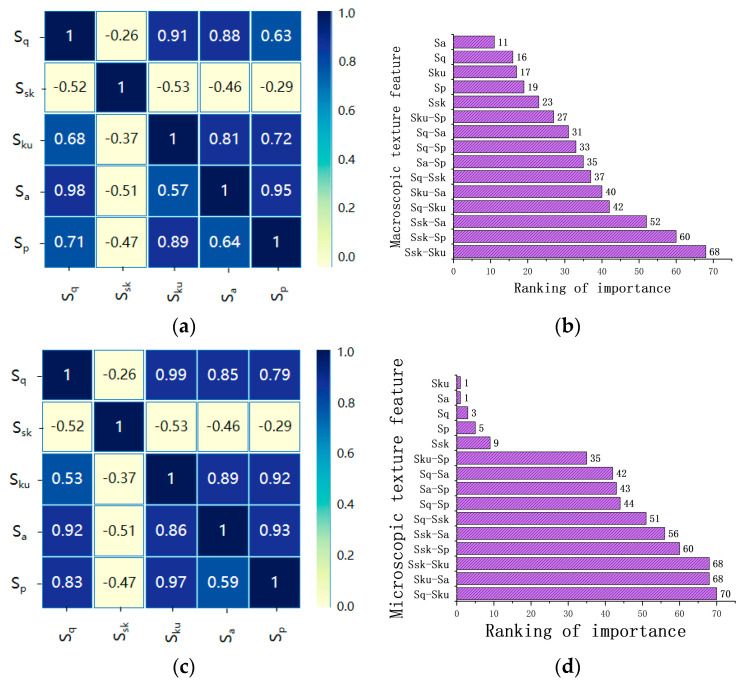
Research on the sensitivity coefficient of morphological parameters. (**a**) Analysis results of resistance sensitivity to contraction. (**b**) Ranking of macro-influence rates. (**c**) Results of the sensitivity analysis of the arc burning coefficient. (**d**) Micro-influence rate ranking.

The influence of a single morphological parameter on electrical linearity is not necessarily a linear superposition when compared to the effects of two or more composite parameters. To prevent deviations in the computational trend of the predictive model, which could lead to non-convergence of final results, an accelerated life test was used to analyze the electrical sensitivity of different composite morphological parameters. The sensitivity coefficients are presented in the form of a heat map, as shown in Figure 9, where darker colors indicate a closer linear correlation between composite morphological parameters and electrical performance. Positive values along the way indicate that the composite parameters enhance the development trends of specific electrical parameters, while negative values indicate a suppressive effect. Figure 9b ranks the importance of the influence of single and composite morphological parameters on electrical performance, visually demonstrating which morphological parameters have a significant impact on electrical parameters.

## 5. Establishment of Electrical Parameter Estimation Model and Parameter Optimization

A single scan probe will generate around 3400 point cloud data. The point cloud dataset of the test object is randomly divided into training set and testing set at a ratio of 80% and 20%, using the testing set to evaluate the predictive performance of the model. Three models are selected for simulation prediction: XGBoost, LightGBM, and CatBoost (Pycharm2023.3.0). See Table 2.

The double sum of squares parameter is used for optimal model comparison, and the performance of the estimated model is judged by three evaluation metrics: the root mean square error (RMSE), mean absolute error (MAE), and coefficient of determination (R^2^). These three common evaluation metrics reflect the error and fitting degree between the model’s predicted values and the actual values. RMSE is negatively correlated with the denoising effect; a smaller RMSE indicates a better denoising effect, and when R^2^ is close to 1, it means it is more similar to the true value. The results of the three models are displayed in Table 3 regarding the dataset containing different dimensional information, with each model estimated using single morphological parameters and composite morphological parameters.

The table above shows the predictive performance of various models on the simulated test set. The results indicate that XGBoost demonstrates a stronger advantage in handling the morphological parameters of electrical contacts. In terms of evaluation metrics, the RMSE and MAE of XGBoost are lower than those of the other two estimation models when dealing with the estimation of single-dimensional morphological parameters, with RMSE of XGBoost being 0.1441 and 0.0806 lower than the other two models, respectively. Meanwhile, R^2^ of XGBoost is also closer to 1 compared to the other estimation models, showing better resistance to interference. Moreover, in the handling results of composite morphological parameters, XGBoost, like the other estimation models, also experiences a significant worsening of its fitting performance; however, the degree of this is relatively less pronounced. Thus, it is proven that XGBoost has significant advantages in both fitting accuracy and resistance to interference.

**Figure 10:**
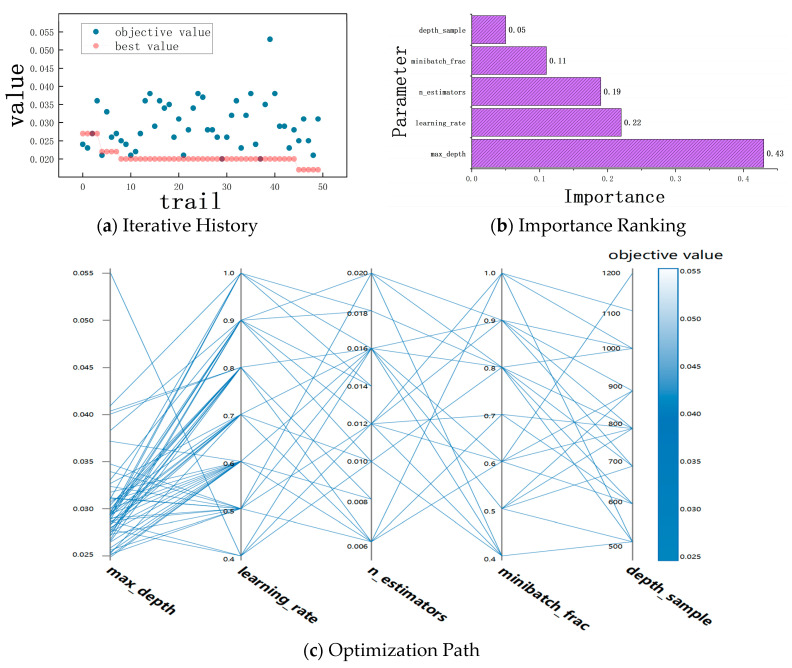
Parameter optimization process.

The introduction of sensitivity coefficients can only characterize the tendency of electrical parameters during morphological changes. In the fitting process with real electrical parameters, various random factors such as different assembly conditions, texture overlay types, and material loss probabilities can affect the classification models like XGBoost, leading to distortion phenomena in the subsequent learning process. To ensure that the estimated key parameters can converge during model iteration, parameter optimization iterations, importance ranking, and parameter space distribution optimization are performed for each tree model, ultimately yielding the optimal spatial distribution solution. The optimization process mainly focuses on optimizing decision tree parameters and certain key parameters: max_depth represents the maximum depth of the decision tree to avoid model overfitting; n_estimators determines the number of base learners to balance model computational efficiency; learning_rate determines the speed at which the model searches for the optimal value; and minibatch_frac and depth_sample control the amount of data processed in each iteration. Figure 10 shows the historical process of parameter optimization iterations, importance ranking, and optimization path.

From the fitting spatial distribution results in Figure 10, the suitable parameters can be easily calculated and fit into the subsequent estimation model with the appropriate spatial distribution parameter values as follows:

Lambda = 0.0013, alpha = 0.0012, depth_sample = 0.6, minibatch_frac = 0.3, n_estimators = 0.9, learning_rate = 0.023, max_depth = 25, min_depth = 2.

**Figure 11:**
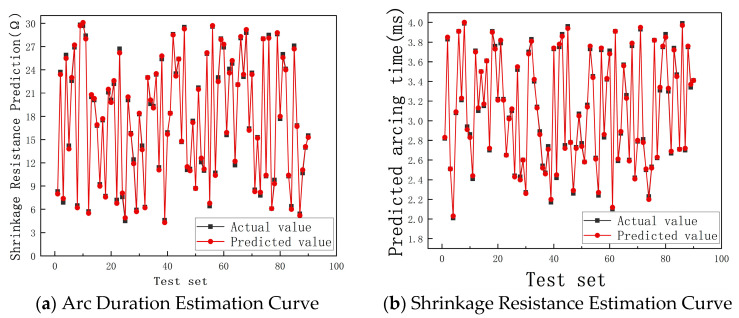
Accuracy estimation of XGBoost.

Under the same data collection conditions, the electrical parameter estimation models based on parameter-optimized XGBoost, LightGBM, and CatBoost were applied to the same morphology dataset. Subsequently, the parameter values of the two typical electrical features, arc time and shrinkage resistance, were separately displayed in Figure 11a,b. The comparison results of the estimated values and the actual values are shown in the following figure, where the black curve represents the actual values from contact measurements, and the red curve represents the estimated electrical values from the models. The closer the two curves are, the better the estimation effect. Any deviation of more than 5% between the predicted values from the model and the actual values is considered a forecasting failure. From the comparative experiments, it can be seen that the estimation accuracy for different electrical parameters of each model is over 90%, with XGBoost, LightGBM, and CatBoost achieving accuracies of 97.2%, 98.8%, and 97.9%, respectively, meeting the requirements for actual production.

## 6. Conclusions

This article builds a non-contact laser-based point cloud collection device for silver contacts, establishing an estimation model for the collected morphological parameters to calculate the electrical performance parameter values under the current surface quality status of the contacts in real time. Additionally, the following objectives have been achieved:

(1) A set of microscopic morphology acquisition devices based on a laser profilometer were designed to address the structural characteristics and installation features of low-voltage electrical contacts. The collected point cloud was processed using a custom mathematical modeling method for point cloud datasets, improving the computational accuracy and the identification of anomalous point clouds. Subsequently, this study analyzed the contour signals in the point cloud dataset, and based on the separation of microscopic and macroscopic texture features from the original contour, an effective weight function robust filtering algorithm was established to further suppress the influence of noise points. This effectively avoided the problem of the neglect of mixed microscopic texture features affecting the estimation accuracy in empirical models, significantly enhancing the operational efficiency of the morphology acquisition device.
(2) Based on the amplitude parameter evaluation method of the micro-surface, the typical morphology parameter indicators of low-voltage contacts are modeled in a numerical way to enhance the reliability and computational consistency of the predictive model. A sensitivity coefficient analysis is established to study the nonlinear impact of composite morphology parameters on electrical performance under the condition of overlapping distributions of more than one morphology parameter, and a correlation analysis table is created, addressing the issue of traditional empirical models only considering a single morphology parameter.
(3) This study uses tests to verify the performance of the model in predicting electrical parameters. Compared with LightGBM and CatBoost, XGBoost exhibits a superior fitting accuracy and anti-interference ability. Subsequently, datasets from multiple contactors were input into the prediction system based on the XGBoost model. The arc time and contraction resistance calculated by the prediction model were compared with the real values. The experimental results demonstrate that the prediction model selected in this study can achieve a 97.2% accuracy in processing these two typical electrical characteristic parameters.

## Figures and Tables

**Table 1:** Morphological parameters.

Parameter	Mathematical Expression	Meaning of Geometric Features
Root mean square deviation of surface topography: $S_{q}$	$\sqrt{\frac{1}{mn}\sum_{i=1}^{m}\sum_{j=1}^{n}S^{2}\left(x_{i},y_{j}\right)}$	Symmetrical distribution of elevation differences
Skewness of surface height distribution: $S_{sk}$	$\frac{1}{mnS_{q}^{4}}\sum_{i=1}^{m}\sum_{j=1}^{n}S^{3}(x_{i},y_{j})$	Reference plane inclination degree
$\mathrm{Surface}\;\mathrm{height}\;\mathrm{gradient}\;\mathrm{steepness}:S_{ku}$	$\frac{1}{mnS_{q}^{4}}\sum_{i=1}^{m}\sum_{j=1}^{n}S^{4}(x_{i},y_{j})$	Surface stability
$\mathrm{Average}\;\mathrm{surface}\;\mathrm{height}:\;S_{a}$	$\frac{1}{mn}\sum_{i=1}^{m}\sum_{j=1}^{n}\left|S(x,y)\right|$	Overall evaluation of roughness
Maximum height of the peak: $S_{p}$	$\underset{(x_{i},y_{j})\mathrm{\epsilon D}}{\max}S(x_{i},y_{j})$	Material pile degree

**Table 2:** Optimal parameters for machine learning models.

Model	Learning Rate	Number of Base Learners	Number of Nodes	Tree Depth
XGBoost	0.1	50	100	7
CatBoost	0.1	100	62	6
LightGBM	0.3	70	60	6

**Table 3:** Comparison of model performance.

Model	Estimation Results for Single Morphology			Estimation Results for Composite Morphology		
	RMSE	MAE	R^2^	RMSE	MAE	R^2^
XGBoost	1.2165	0.8928	0.9877	2.2106	1.1231	0.9700
CatBoost	1.3606	1.0211	0.9482	2.6068	1.5247	0.8740
LightGBM	1.1359	0.9413	0.9751	2.4129	1.1523	0.9277

## Data Availability

The original contributions presented in this study are included in the article. Further inquiries can be directed to the corresponding author.
